# The impact of different artificial disc heights during total cervical disc replacement: an in vitro biomechanical study

**DOI:** 10.1186/s13018-020-02157-9

**Published:** 2021-01-06

**Authors:** Xiao-Fei Wang, Yang Meng, Hao Liu, Bei-Yu Wang, Ying Hong

**Affiliations:** 1 Department of Orthopaedic Surgery, West China Hospital, Sichuan University, No. 37 Guo Xue Xiang, Chengdu, 610041 Sichuan China; 2 Department of Anesthesia and Operation Room/West China School of Nursing, West China Hospital, Sichuan University, No. 37 Guo Xue Xiang, Chengdu, 610041 Sichuan China

**Keywords:** Total disc replacement, Biomechanics, Cervical facet joint, Implant height

## Abstract

**Background:**

The principles of choosing an appropriate implant height remain controversial in total cervical disc replacement (TDR). By performing an in vitro biomechanical study and exploring the biomechanical impact of implant height on facet joint and motion function, the study aimed to offer valid proposals regarding implant height selection during TDR.

**Methods:**

A total of 6 fresh-frozen male cadaveric cervical spines (C2–C7) with 5 mm intervertebral disc height at C5/6 level were enrolled in the study. Specimens with the intact condition and with different height artificial discs were tested. Facet joint pressures and range of motion under each condition were recorded using a specialized machine.

**Results:**

The artificial disc heights that were involved in this study were 5 mm, 6 mm, and 7 mm. The range of motion decreased along with the increment of implant height, while facet joint pressure showed an opposite trend. Specimens with a 5 mm implant height could provide a similar range of motion (11.8° vs. 12.2° in flexion-extension, 8.7° vs. 9.0° in rotation, 7.9° vs. 8.2° in lateral bending) and facet joint pressure (27.8 psi vs. 25.2 psi in flexion, 59.7 psi vs. 58.9 psi in extension, 24.0 psi vs. 22.7 psi in rotation, 32.0 psi vs. 28.8 psi in lateral bending) compared with intact specimens. Facet joint pressure of specimens with 6 mm implant height (≥ 1 mm in height) increased during flexion at the C5–6 segment (30.4 psi vs. 25.2 psi, *P* = 0.076). However, specimens with 7 mm implant height (≥ 2 mm in height) showed a significant reduction in motion (9.5° vs. 12.2° in flexion-extension, *P* < 0.001) and increment of facet joint pressure at C5–6 segment (44.6 psi vs. 25.2 psi in flexion, 90.3 psi vs. 58.9 psi in extension, *P* < 0.0001) and adjacent segments.

**Conclusions:**

This study suggested that an appropriate artificial disc height can achieve near-normal biomechanical properties and is recommended. We should be very cautious when using artificial discs ≥ 1 mm in height compared to normal. However, implants ≥ 2 mm in height compared to normal significantly increased the facet joint pressure and decreased the range of motion; therefore, it should not be used in clinical practice.

## Introduction

Total cervical disc replacement (TDR) is increasingly being used in clinical practice with the widespread concept of non-fusion surgery. Compared with anterior cervical discectomy and fusion (ACDF), TDR has advantages in maintaining the biomechanical condition of the cervical spine, reconstructing range of motion (ROM) at index levels, and reducing compensatory increasing of ROM at adjacent levels [1–3]. In addition, patients who undergo TDR do not require long-time limitation of their necks, thus accelerating the rehabilitation process [4]. Hu et al. carried out a meta-analysis and showed that TDR is non-inferior to ACDF in some ways such as neurological functional recovery, NDI (Neck Disability Index) scores, and JOA (Japanese Orthopaedic Association) scores and is even better than ACDF in terms of post-surgical complications, reoperation rates, and patient satisfaction [5]. Therefore, using TDR to treat cervical degenerative disc disease could achieve significant clinical effects.

Choosing an appropriately sized artificial cervical disc is an important process during TDR. In clinical practice, we may encounter a situation wherein both prostheses (for example, 5- and 6-mm-high prostheses) fit the index intervertebral disc space well according to the intraoperative trailing process. However, it remains controversial how to appropriately select the most suitable height of the artificial disc under this circumstance. By performing a clinical study, Rong et al. considered that a higher artificial disc is better for relieving neurological deficit symptoms by enlarging the volume of the intervertebral foramen and increasing the stability of the index segment [6]. Unlike their opinion, a finite element study (FE study) carried out by Yuan et al. concludes that the lower implant could provide near-normal biomechanical properties, while an oversized one would lead to marked changes of the cervical biomechanical features [7].

While being limited to the confounding factors, such as measuring errors, clinical studies may not be able to elucidate the direct relationship between implant height and clinical outcomes. In addition, the results from FE studies may be affected by different modeling parameters; thus, FE studies may not emulate a physiological condition of the cervical spine. As for biomechanical studies, cadaveric cervical spine specimens are used for testing, and a specialized recorder is used to collect data. Therefore, biomechanical studies could fill the gaps in clinical and FE studies. The cervical facet joint, one of the components of the three-joint complex, is important to load transmission and movement of the cervical spine [8]. However, the biomechanical impacts of different artificial disc heights on facet joints during TDR have not been reported. Therefore, we performed an in vitro biomechanical study using human cadaveric cervical spines to observe the biomechanical impact of different implant heights on facet joint pressure. We hypothesized that a higher artificial disc would result in larger facet joint pressure and lower range of motion, thus should not be used during surgery.

**Fig. 1:**
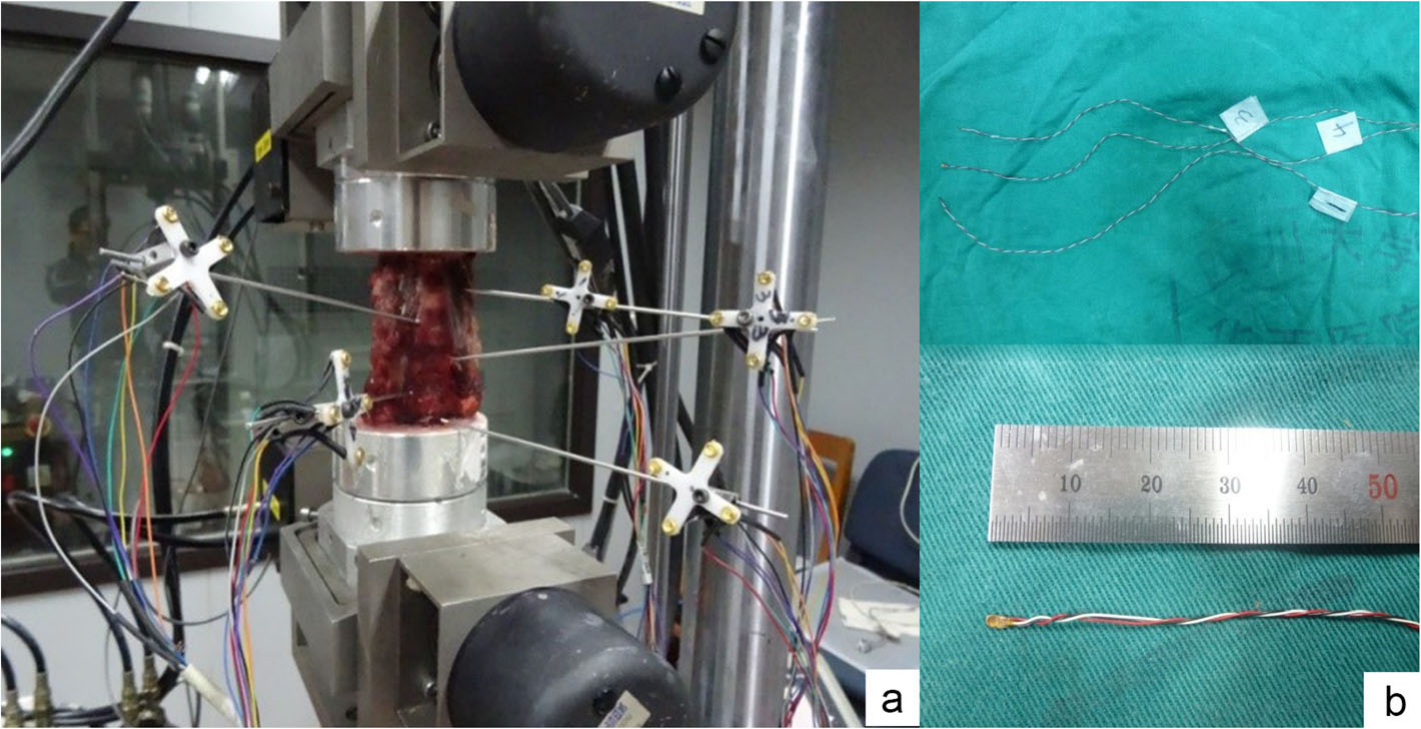
Lateral view of an intact specimen. **a** The specimen was fixed in a physiological curvature and inserted with optical markers. **b** The precision pressure measuring sensors that were inserted into the facet joint gaps

Different heights of artificial cervical discs, the Pretic-I cervical disc (Trauson, China) (Fig. 1), was used in this biomechanical study. Previously, we carried out a series of biomechanical studies using the Pretic-I cervical disc, and we found in terms of IDP and ROM, the Pretic-I cervical disc is non-inferior to Discover and Prestige-LP cervical disc. In addition, our clinical study revealed Pretic-I cervical disc could achieve satisfactory clinical results [9]. By analyzing the results of this study, we also offered valid proposals regarding the selection of the appropriate implant height during TDR.

## Methods

### Specimen preparation

This study was an in vitro biomechanical study and was approved by the Institutional Review Board of West China Hospital. Six fresh-frozen cadaveric cervical spines (C2–C7) that came from donors were included in this biomechanical test. The intervertebral disc heights that were measured on the lateral radiographs were 4.9 mm, 5.2 mm, 5.3 mm, 5.2 mm, 5.1 mm, and 5.2 mm, respectively. Surrounding soft tissues and muscles of the six specimens were carefully dissected. The ligamentous structures, intervertebral discs, and facet joint capsules were preserved. Radiographs and bone scanning were tested to exclude specimens with obvious flaws such as fractures, deformities, tumors, osteoporosis, or disc degeneration (osteophytes, disc space narrowing, or facet hypertrophy).

### Biomechanical testing

Before biomechanical testing, the proximal (C2) and distal (C7) ends of each specimen were embedded in polymethylmethacrylate in cylindrical aluminum fixtures (C7 vertebral segment was reinforced by partially inserting three perpendicular screws). A multi-degree of freedom servo-hydraulic testing system (MTS Bionix 858, MTS Corporation, Minneapolis, MN, USA) was used in this study. This platform has been used to perform cadaveric biomechanical studies in the cervical [10, 11] or lumbar [12–14] spine and could provide reproducible results because it applies uniformly distributed load through the specimen despite the point of load application [15]. We used this device in our previous cadaveric studies to explore biomechanical features including the range of motion and intervertebral disc pressure (IDP) of the cervical spine under different conditions, and we got validated results [16, 17].

A load-control protocol was used and a 75N follower load was applied to stimulate the physiological compressive loads. To induce normal movement of the cervical spine (flexion, extension, lateral bending, and axial rotation), we applied pure moments of 2.0 Nm maximum with a constant rate of 0.2 Nm/s about the 3 primary anatomical axes.

**Fig. 2:**
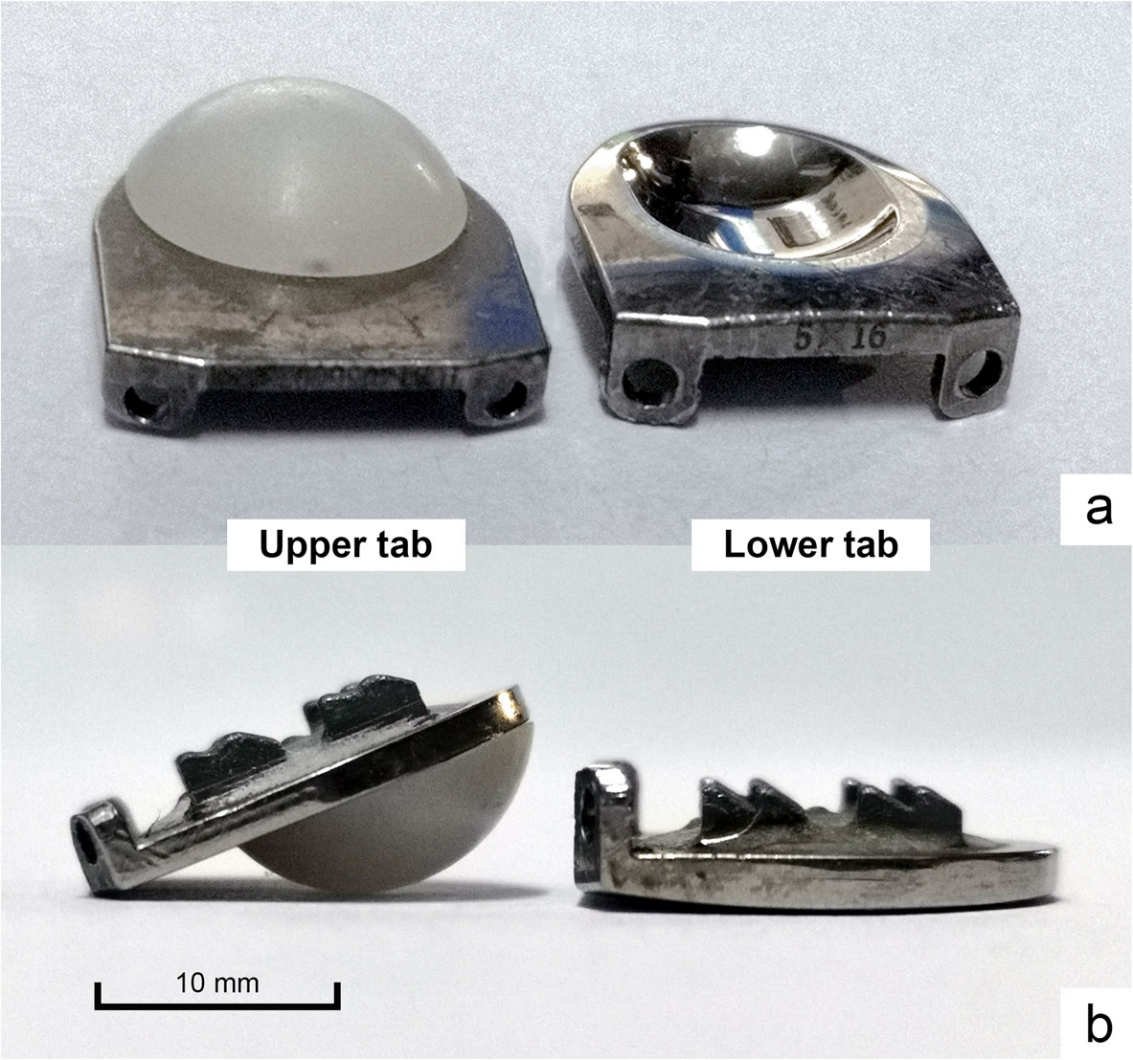
The Pretic-I cervical disc prosthesis is a semi-constrained prosthesis with a ball-trough design **a**. The superior and inferior plates of Pretic-I are designed with an arcuate surface **b**

To evaluate the segmental ROM of the specimens at the surgical level, we used an optical tracking system (Polaris Northern Digital Incorporation, Ontario, Canada). For each vertebral body from C4 to C7, a Kirschner pin connecting four optical markers was inserted to the lateral part. To record the facet joint pressure, we incised the right facet joints at C4–7 along the articular surfaces and inserted Precision micro pressure sensors (Precision Measurement Company, Ann Arbor, Michigan, USA, connected with a signal collector) into joint gaps, as Cripton et al. [18] and Patel et al. [19] described before (Fig. 2). Delicate manipulations were taken to avoid oversized incisions. We took radiographs to make sure the micro pressure sensors were in facet joint gaps.

**Fig. 3:**
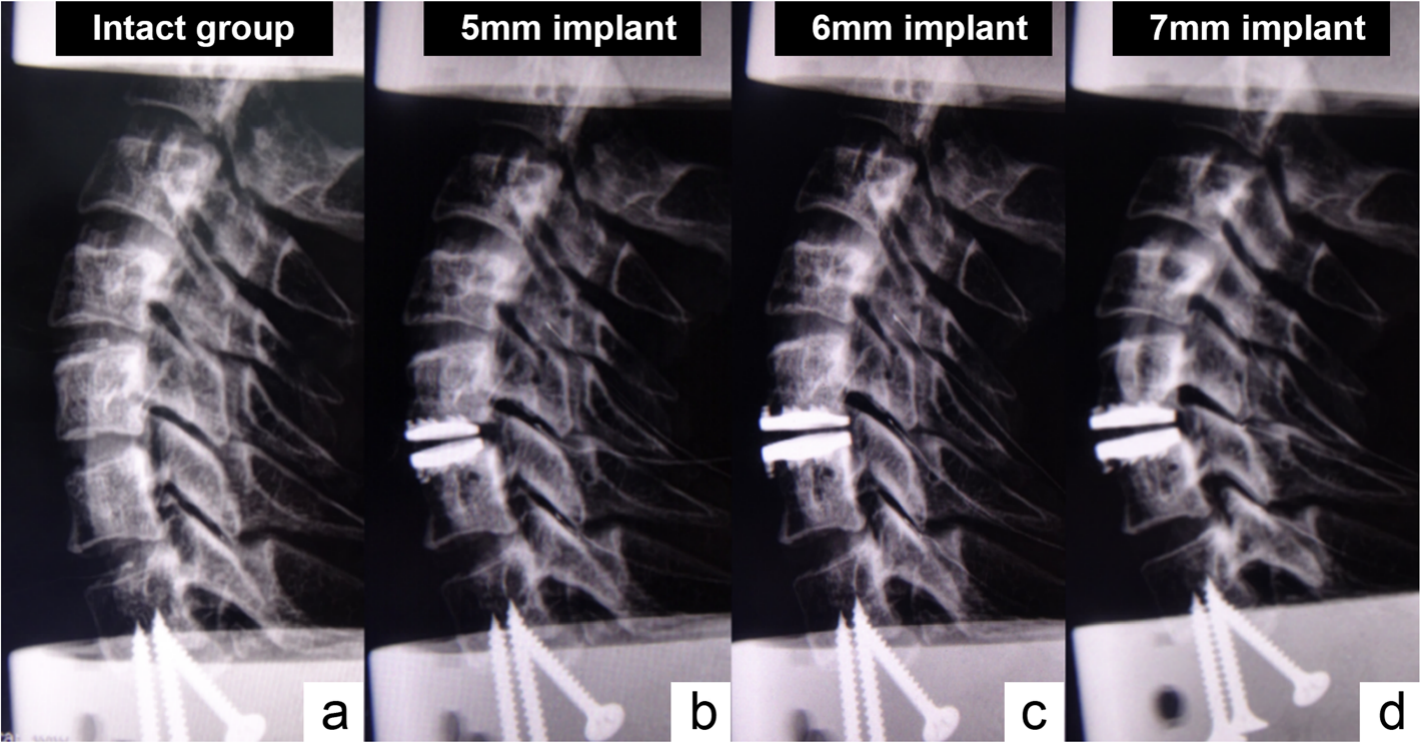
The lateral radiographs of a specimen with the intact condition **a**, 5-mm implant **b**, 6-mm implant **c**, and 7-mm implant **d**. The facet joints at C5–6 were gradually stretched with the increment of implant height, and the articular surfaces were not parallel with a 7-mm-high artificial disc inserted **d**

We first tested the biomechanical features of six intact specimens and recorded related data. Subsequently, we inserted different heights of Pretic-I artificial discs in an ascending order (5 mm, 6 mm, and 7 mm) followed by the standard surgical procedure to make sure the implants were in the correct position (on coronal radiographs, the implant is axis-symmetric without coronal titling; on lateral radiographs, the implant is fully inserted into the intervertebral disc space without migration). Then, we recorded the biomechanical data with each implant height, respectively. The length of Pretic-I was 16.5 mm. The other dimensions of Pretic-I (Fig. 1) were described in detail before [20]. Each test was repeated for three loading cycles, and the data from the third loading cycle was used for analysis. The six intact specimens were defined as group 1, and specimens with 5 mm, 6 mm, or 7 mm implant heights were defined as group 2, group 3, or group 4, respectively (Fig. 3).

### Statistical analysis

Statistical analysis was performed using SPSS software for Windows version 21.0 (SPSS Inc., Chicago, IL, USA). Presented in the form of “Average ± standard error (SD),” we used one-way ANOVA analysis to determine whether or not the outcomes were significantly different among the 4 groups. The values between groups were analyzed by the Tukey test (equal variance) or the Games-Howell test (unequal variance). A value of *P* < 0.05 was considered statistically significant.

## Results

### Intervertebral disc height

The mean intervertebral disc height (measured on the lateral radiograph) of group 1, group 2, group 3, and group 4 was 5.2 ± 0.1 mm, 7.1 ± 0.1 mm, 8.4 ± 0.2 mm, and 9.6 ± 0.1 mm, respectively. There was a significant difference among all groups (*P* < 0.05).

### Range of motion at C5–6 segment

**Fig. 4:**
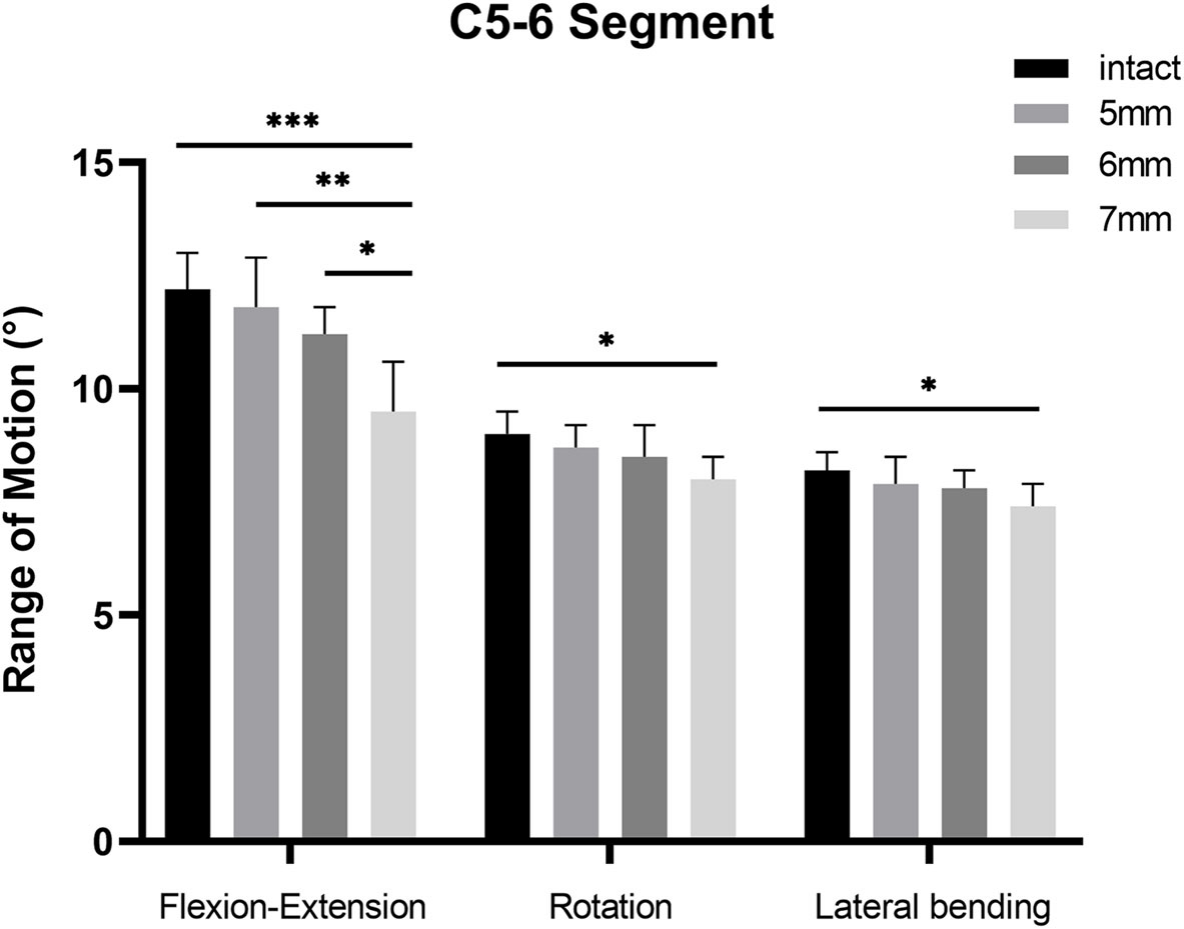
The range of motion of each group at the C5–6 level

The results are shown in Fig. 4. The ROM at the surgical level decreased along with the increment of implant height. Both group 2 (11.8 ± 1.1° in flexion-extension, 8.7 ± 0.5° in rotation, 7.9 ± 0.6° in lateral bending) and group 3 (11.2 ± 0.6° in flexion-extension, 8.5 ± 0.7° in rotation, 7.8 ± 0.4° in lateral bending) showed similar ROM in all directions compared with group 1 (12.2 ± 0.8° in flexion-extension, 9.0 ± 0.5° in rotation, 8.2 ± 0.4° in lateral bending) (*P* > 0.05). However, group 4 showed a significantly lower ROM during flexion-extension (9.5 ± 1.1°) compared with the other three groups (*P* < 0.05). The ROM during lateral bending (7.4 ± 0.5° vs. 8.2 ± 0.4°, *P* < 0.05) and axial rotation (8.0 ± 0.5° vs. 9.0 ± 0.5°, *P* < 0.05) of group 4 was significantly lower compared with that of group 1.

### Facet joints pressure at C5–6 segment

**Fig. 5:**
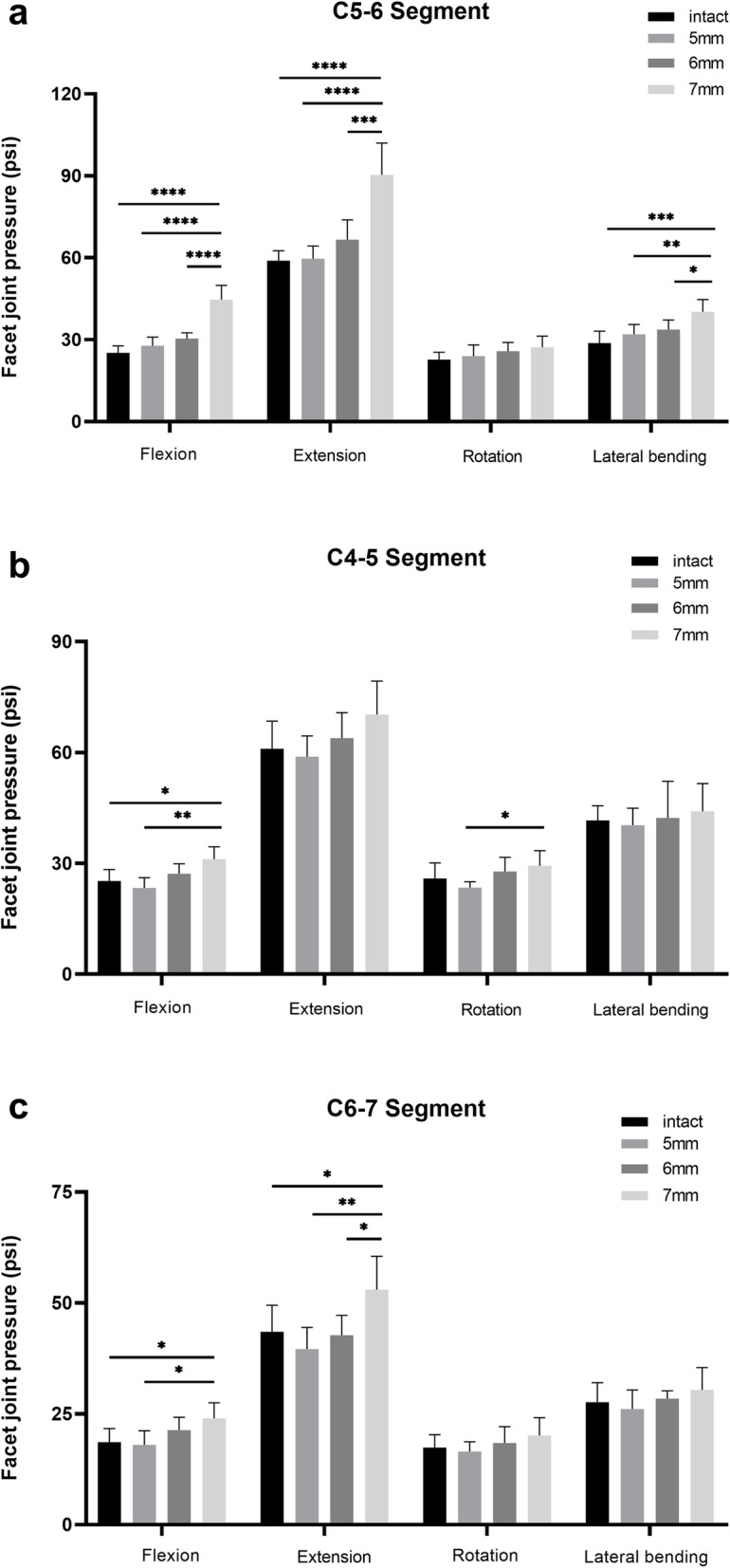
The mean facet joint pressure of each group at C5–6 **a**, C4–5 **b**, and C6–7 **c** during flexion, extension, rotation, and lateral bending

The results are shown in Fig. 5a. The facet joint pressures of group 2 (27.8 ± 3.1 psi in flexion, 59.7 ± 4.6 psi in extension, 24.0 ± 4.1 psi in rotation, 32.0 ± 3.6 psi in lateral bending) were higher than those of group 1 (25.2 ± 2.5 psi in flexion, 58.9 ± 3.7 psi in extension, 22.7 ± 2.7 psi in rotation, 28.8 ± 4.3 psi in lateral bending) at the C5–6 segment, while the difference was not statistically significant (*P* > 0.05). Facet joint pressures increased along with the increment of implant height in all directions. When comparing group 3 with group 1, we found that facet joint pressures were higher during flexion (30.4 ± 2.1 psi vs. 25.2 ± 2.5 psi, *P* = 0.076). In addition, the facet joint pressures of group 4 were higher than those of other groups in flexion (44.6 ± 5.3 psi), extension (90.3 ± 11.7 psi), and lateral bending (40.2 ± 4.5 psi) (*P* < 0.05).

### Facet joints pressure at the C4–5 segment

The results are shown in Fig. 5b. The facet joint pressures of group 2 (23.3 ± 2.8 psi in flexion, 58.9 ± 5.6 psi in extension, 23.4 ± 1.6 psi in rotation, 40.3 ± 4.6 psi in lateral bending) were lower than those of group 1 (25.2 ± 3.1 psi in flexion, 61.0 ± 7.5 psi in extension, 25.9 ± 4.2 psi in rotation, 41.6 ± 4.0 psi in lateral bending) without a significant difference (*P* > 0.05). The was a trend for facet joint pressures to increase along with implant heights in group 2, group 3, and group 4. When comparing group 4 with group 1, significant differences in facet joint pressures were found during flexion (31.1 ± 3.4 psi vs. 25.2 ± 3.1 psi, *P* < 0.05). When comparing group 4 with group 2, significant higher pressure was found during flexion (31.1 ± 3.4 psi vs. 23.3 ± 2.8 psi, *P* < 0.01) and rotation (29.3 ± 4.1 psi vs. 23.4 ± 1.6 psi, *P* < 0.05), and the difference was apparent during extension (70.3 ± 9.0 psi vs. 58.9 ± 5.6 psi, *P* = 0.063).

### Facet joints pressure at C6–7 segment

The results are shown in Fig. 5c. Similarly, with the increasing of implant heights, the facet joint pressures at C6–7 were also adding. Although pressures of group 2 (18.0 ± 3.2 psi in flexion, 39.6 ± 4.9 psi in extension, 16.5 ± 2.2 psi in rotation, 26.1 ± 4.3 psi in lateral bending) were not as high as those of group 1 (18.6 ± 3.1 psi in flexion, 43.5 ± 6.0 psi in extension, 17.4 ± 2.9 psi in rotation, 27.6 ± 4.4 psi in lateral bending), we did not find a significant difference between these two groups (*P* > 0.05). However, in comparison to group 4, the facet joint pressures of group 1 (18.6 ± 3.1 psi vs. 24.0 ± 3.5 psi, *P* < 0.05) and group 2 were much lower in flexion (18.0 ± 3.2 psi vs. 24.0 ± 3.5 psi, *P* < 0.05). Meanwhile, during extension, the facet joint pressures of group 4 (53.0 ± 7.5 psi) were significantly higher than those of other groups (*P* < 0.05).

### Facet joints angle at C5–6 segment

**Fig. 6:**
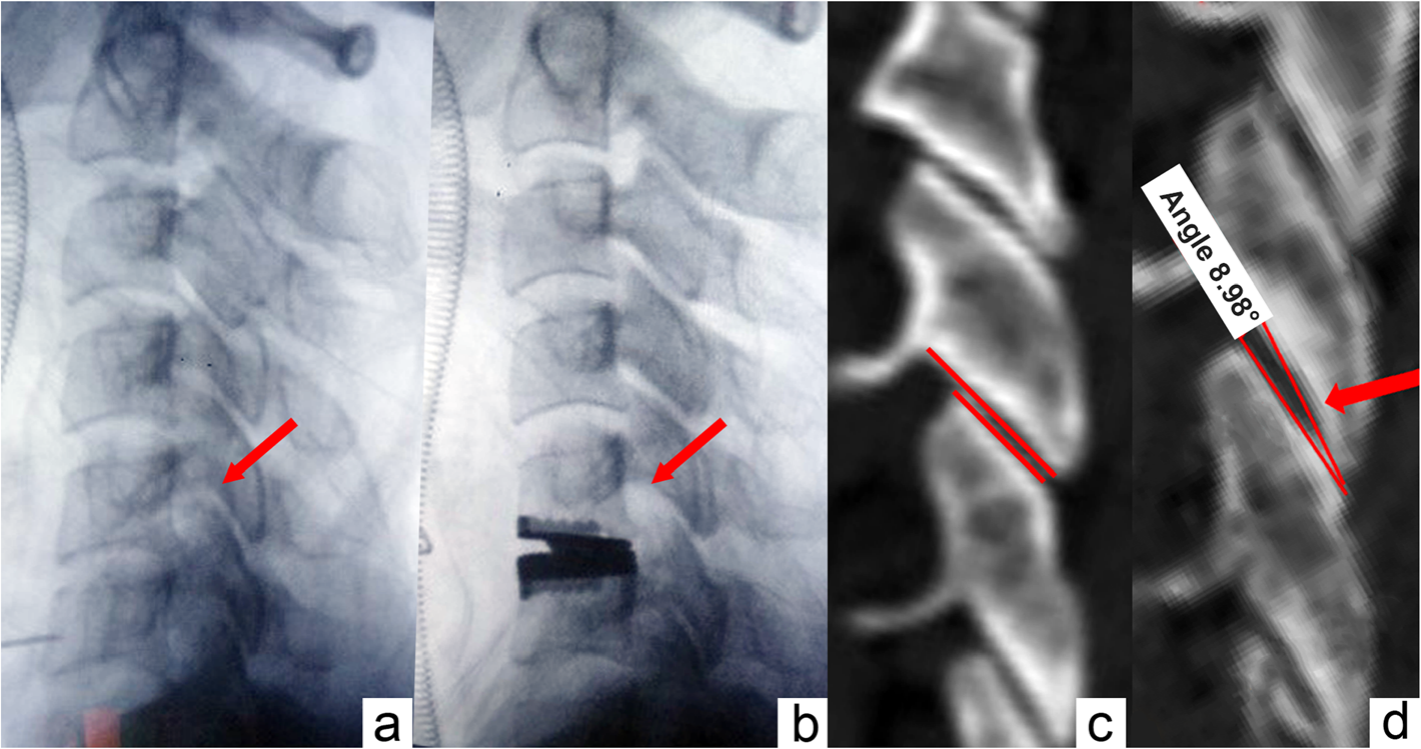
In clinical practice, we may encounter the situation that the facet joint gaps at the index level were stretched by an oversized artificial disc **a**, **b** the facet joints are labeled with arrows). Besides, the normal articular surfaces were parallel which could be observed in CT scan **c**, and we found that with the increment of intervertebral height, the articular surfaces at index level would form a certain angle **d** the angled facet joint is labeled with an arrow)

Facet joint angles were measured as shown in Fig. 6. The facet joint angles of group 1, group 2, group 3, and group 4 were 0.8 ± 0.5°, 1.7 ± 0.8°, 4.0 ± 1.0°, and 9.5 ± 1.3°, respectively. There was no difference between group 1 and group 2 (*P* = 0.459). Except for this, there was a significant difference among other groups (*P* < 0.001).

## Discussion

Implant height selection is an important process during TDR. However, there is no consensus about how to choose the most appropriate height of prosthesis. A higher artificial disc could enlarge the volume of the intervertebral foramen, which is good for relieving neurological deficit symptoms. A higher implant could also increase the stability of the index segment by filling intervertebral disc space. On the other hand, overstretching of intervertebral disc space by an oversized artificial disc may lead to increments of stress distribution on facet joints [7] and complications such as heterotopic ossification [21]. Furthermore, some authors hold the view that physiological biomechanical properties, at index as well as adjacent levels, could be altered by a larger implant [7, 22].

Rong et al. [6] suggested that a higher implant height may be favorable. In the study, they enrolled 34 patients who underwent TDR from 2008 to 2014. In these patients, 5- and 6-mm-high prostheses were used; 17 of them were inserted with 5-mm-high prostheses, and the remaining 17 patients received 6-mm prostheses. By assessing neurological functional scores, such as the JOA score and the NDI score, they found patients with 6-mm prostheses achieved better clinical outcomes, and they associated this result to the increment of intervertebral foremen size. On the contrary, Yuan et al. [7] suggested that lower height could satisfactorily provide near-normal biomechanical properties. They carried out a FE study to explore the influence of the height of Prestige-LP cervical disc on the cervical biomechanics and found that, compared with intact conditions, the bone-implant interface stress and intradiscal disc pressure of the model with larger sized prostheses were increased, and subsidence of prosthesis could be induced.

The most commonly used implant height in total cervical disc replacement was 5 mm [23]. Therefore, we enrolled specimens with 5 mm intervertebral disc height in this study, and we recorded the facet joint pressure and segmental ROM with intact conditions and with implants of 5 mm height (the same as normal), 6 mm height (≥ 1 mm height than normal), and 7 mm height (≥ 2 mm height than normal). We aimed to provide suggestions for surgeons regarding how to choose the most appropriate implant height during TDR.

Cervical facet joints play an important part in the load transmission of the cervical spine. Pal and Routal [24] carried out a morphological study and reported that roughly 23% of the axial compressive load is transmitted by the facet joints in the cervical spine. Previously, few studies showed that the implant height could influence facet joint pressure after TDR. Womack et al. [22] studied the impact of implant height on facet joints after TDR using Prodisc-C prosthesis by performing a FE study, and they found that facet joint pressures revealed a negative relationship with implant height, and the impact of implant height on facet joints was more significant in index levels than in adjacent levels. In contrast with their results, Yuan et al. [7] revealed that with the increment of implant heights, the facet joint pressure at the index level decreased, while the pressure would increase at adjacent levels. In addition, a 7-mm-high prosthesis would lead to significant change. However, the results of FE studies could be affected by modeling and parameters; thus, it might not reflect the physiological condition of the cervical spine as biomechanical studies could.

In our study, we observed that the facet joint pressure was altered after implantation of artificial discs, and with higher implants, the facet joint pressure at the index level was significantly changed during flexion-extension. Furthermore, we observed a trend for facet joint pressures at adjacent levels that increased along with the implant height. With regard to a 7 mm implant height, the facet joint pressure at the index level increased by 77% during flexion, 53% during extension, and 40% during lateral bending (all with a significant difference). The facet joint pressure during flexion-extension at adjacent levels also increased compared with the intact specimens. Our results could be explained by the following points: Firstly, a cadaveric radiological study by Liu et al. [25] revealed that at each 1-mm incremental increase in disc space at C5–C6 levels the articulation overlap decreased by approximately 7% and the facet joint space increased approximately 0.8 mm. Secondly, by taking lateral radiographs, we found that with the increase of implant height, the facet joints at the index level were gradually stretched (Figs. 3 and 6a, b). The angle between the articular processes at the index level increased while the overlap surface of facet joints was decreased, and the articular surfaces at the index level were not parallel (Fig. 3 and 6c, d). In other words, with the increment of intervertebral disc height, the composition of facet joints changed to “line-to-surface” from “surface-to-surface.” In accordance with physical laws, there is a negative correlation between the intensity of pressure and the contact area, which means that with the reduction of facet joint overlap area, the facet joint pressure increases. Moreover, Pretic-I cervical disc is designed with a semi-constrained structure with a backward location of rotation center, and lordosis of the cervical spine increases along with the implant height; therefore, the pressure of posterior structures of the cervical spine would also escalate.

The disadvantages of an oversized prosthesis could also be seen in terms of segmental ROM. Our results showed that specimens with a 7-mm prosthesis achieved a significantly lower ROM compared with specimens of other implant heights and intact specimens, which coincide with those of Yuan’s FE study, in that the 5- and 6-high prostheses could preserve well the physiological ROM, while the 7-mm-high prosthesis significantly reduces the motion at C5–6 during flexion-extension and lateral bending [7]. As we described early, the angle between the articular processes at the index level increased and the overlapping pattern of facet joints at the index level changed to line-to-surface with the increasing intervertebral disc height. Furthermore, the facet joints at the index level gradually stretched. Generally, the imbricate arrangement-cervical facet joints played an important part in limiting and guiding the cervical spine movement. The normal overlap of articular surfaces of facet joints contributes to the spinal ROM in six directions. Therefore, the altered line-to-surface structure mode may hinder the normal function of facet joints to move along with the vertebral body as a whole, and the over-distraction of the facet capsule or other soft-tissue restraints may limit intervertebral motion. Li et al. [26] enrolled 160 patients who underwent TDR using Prestige-LP cervical disc and divided these patients into 3 groups according to the postoperative intervertebral disc height (< 6 mm, 6–8 mm, and > 8 mm). With an average of 30 months of follow-up, they found that patients with a 6–8 mm postoperative intervertebral disc height achieved a 20% higher segmental ROM at index level compared to other groups. They showed that a 6–8 mm postoperative intervertebral disc height may maintain the disc height in physiological conditions, and thus could achieve better clinical outcomes. All these provide evidence for not choosing an oversized prosthesis (e.g. prosthesis with 7 mm height).

In this paper, we found that specimens with a 5 mm height (an appropriate height) prosthesis maintained a similar facet joint pressure compared with intact specimens. There is a trend for facet joint pressure to increase along with the implant height, and a 7 mm implant height (≥ 2 mm height than normal) significantly increased the facet joint pressures. Although increments of intervertebral height could enlarge the volume of the intervertebral foramen and thus relieve neurological symptoms, it could also increase the risk of post-surgical complications such as facet joint subluxation, arthritis, and neck pain. Therefore, according to the results of our study, prosthesis with ≥ 2 mm height than normal should not be used during TDR.

There are also some limitations in this study, which we tried out best to minimize their impacts on our results. Firstly, the potential impacts of sequential and repeated testing on stabilizing structures such as facet capsule may exist. In our study, the number of cadavers was limited. Therefore, we could only get results of different implant heights by performing a self-control study through sequential and repeating testing. However, we took several measures to reduce the impacts of testing on stabilizing structures. For instance, we inserted the implants in an ascending order, so that the facet capsule would be stretched gradually. Inserting implants in an ascending order could help facet capsules keep their tension when inserting a higher artificial disc and minimize the impacts on these soft tissues. Next, we dealt with the cadavers carefully and gently to avoid unnecessary damage to stabilizing structures. We cut a small incision on the facet capsule for each cadaver and used micro-sensors (as we described above, each sensing element is a 3 × 1.5 mm^2^ area) to minimize the damage. Also, we set moderate parameters (a 75N follower load and 2.0 Nm pure moment) in our study to avoid overstretching of facet capsule during testing. The second limitation is that one-side sensors may affect the biomechanics of the cervical spine and facet joints. Therefore, the results of our study should be interpreted with caution. Another limitation is that this study was performed using the load-control protocol, which has little ability to represent the adjacent kinematics [27]. However, the adjacent kinematics and adjacent degeneration always are big concerns for TDR. Therefore, future studies using the displacement-control protocol or hybrid protocol are needed to better understand the adjacent kinematics with different implant heights.

## Conclusion

An appropriate height artificial disc can achieve near-normal facet joint load transmission and ROM. Therefore, it is recommended in TDR. Implants ≥ 1 mm in height compared to normal should be used with caution because it increased the facet joint force during flexion. However, implants ≥ 2 mm in height compared to normal significantly increased the facet joint pressure and decreased the ROM; therefore, it should not be used in clinical practice.

## Data Availability

Datasets are available from the corresponding author on a reasonable request.
